# Functional Genetic Variant in ATG5 Gene Promoter in Acute Myocardial Infarction

**DOI:** 10.1155/2020/9898301

**Published:** 2020-04-21

**Authors:** Yexin Zhang, Xiaohui He, Jiarui Li, Wentao Yang, Yinghua Cui, Shuchao Pang, Haihua Wang, Bo Yan

**Affiliations:** ^1^Department of Medicine, Shandong University School of Medicine, Jinan, Shandong 250012, China; ^2^Division of Cardiology, Affiliated Hospital of Jining Medical University, Jining Medical University, Jining, Shandong 272029, China; ^3^Shandong Provincial Key Laboratory of Cardiac Disease Diagnosis and Treatment, Affiliated Hospital of Jining Medical University, Jining Medical University, Jining, Shandong 272029, China; ^4^The Center for Molecular Genetics of Cardiovascular Diseases, Affiliated Hospital of Jining Medical University, Jining Medical University, Jining, Shandong 272029, China; ^5^Shandong Provincial Sino-US Cooperation Research Center for Translational Medicine, Affiliated Hospital of Jining Medical University, Jining Medical University, Jining, Shandong 272029, China

## Abstract

Coronary artery disease (CAD) including acute myocardial infarction (AMI) is an inflammatory and metabolic disease mainly caused by atherosclerosis. Dysfunctional autophagy has been associated with abnormal lipid metabolism and inflammation. In previous studies, we have reported altered autophagic activity in AMI patients. As autophagy-related protein 5 (ATG5) is a core protein in autophagy, we speculated that altered ATG5 level may contribute to CAD and AMI development. In this study, the promoter of the ATG5 gene was genetically and functionally investigated in large groups of AMI patients (*n* = 378) and ethnic-matched healthy controls (*n* = 386). The results showed that a total of 15 genetic variants including 6 single-nucleotide polymorphisms (SNPs) in the ATG5 gene promoter were found in this study population. A novel deletion variant (g.106326168_70delTCT) and an SNP [g.106325757C > G (rs190825454)] were found in one 66-year-old male patient with non-ST-segment elevated AMI, but in none of controls. In cultured HEK-293 and H9c2 cells, the deletion variant significantly decreased the transcriptional activity of the ATG5 gene promoter (*P* < 0.01). In contrast, the genetic variants either identified only in controls or found in both AMI patients and controls did not affect the transcriptional activity of the ATG5 gene promoter (*P* > 0.05). Furthermore, an electrophoretic mobility shift assay showed that the deletion variant evidently affected the binding of a transcription factor. Therefore, the genetic variant identified in AMI may affect the activity of the ATG5 gene promoter and change the ATG5 level, contributing to AMI as a rare risk factor.

## 1. Introduction

Coronary artery disease (CAD) including acute myocardial infarction (AMI) is an inflammatory and metabolic disease, which is mainly caused by atherosclerosis. Abnormal lipid metabolism and inflammation play critical roles in the initiation and progression of atherosclerosis and its complications [1, 2]. Although genome-wide association studies have identified more than 60 genetic loci for CAD and AMI, these collective genetic loci could explain only <10% of cases [3]. Therefore, genetic causes and molecular mechanisms for CAD and AMI remain to be investigated and elucidated.

Autophagy is an evolutionally conserved process in cells to deliver cytoplasmic contents to lysosomes for degradation. Human studies and animal experiments have demonstrated that autophagy plays essential roles in many physiological processes, such as maintaining cellular homeostasis, regulating cell death and survival, aging, lipid metabolism, immune response, and inflammation [4–6]. Dysfunctional autophagy has been implicated in a wide range of human diseases, including neurodegenerative diseases, metabolic syndrome, cardiovascular diseases, and cancers [7, 8]. In the cardiovascular system, autophagy functions in all cell types and is essential to preserve cardiovascular structure and function under baseline conditions [9]. As optimal autophagic activity is critical to cardiovascular homeostasis and function, excessive or insufficient levels of autophagic flux may contribute to the development of cardiovascular diseases, such as atherosclerosis, cardiomyopathy, CAD, and AMI [7, 10].

To date, more than 40 autophagy-related (ATG) proteins have been identified and ATG5 is a critical core protein for autophagy [11]. During the process of autophagosome formation, ATG5 is first formed, a conjugate with ATG12, by the activation of ATG7 (E1-like enzyme) and ATG10 (E2-like enzyme). The ATG5-ATG12 conjugate then binds to membrane-associated ATG16L, thereby forming the ATG16L complex. The ATG5-ATG12-ATG16L complex functions as an E3 ligase to facilitate the covalent conjugation of microtubule-associated protein light chain 3 (LC3)-phosphatidylethanolamine [12–14]. Mice deficient for ATG5 appear almost normal at birth but die within 1 day of delivery [15]. Mice with cardiac-specific ATG5 deletion are born normally and exhibit significant cardiac dysfunction at the age of 10 months [16, 17]. In contrast, ubiquitous overexpression of ATG5 extends median lifespan of mice [18]. Moreover, ATG5 deficiency-mediated autophagy contributes to cardiac inflammation and injury by increasing NF-*κ*B activity in macrophages [19]. In mice with a macrophage-specific ablation of ATG5, P62 deficiency increases atherosclerotic plaque burden, indicating ATG5 involvement in atherosclerosis formation [20].

We have previously reported altered LC3 gene expression levels in CAD and AMI patients [21]. Several genetic variants have been identified in the ATG7 and LC3 gene in AMI patients [22, 23]. As ATG5 is one of core proteins for autophagy, we postulated that dysregulated ATG5 gene expression may lead to altered autophagic activity, playing an important role in the development of CAD and AMI. In this study, we genetically and functionally analyzed the promoter of the ATG5 gene in a large cohort of AMI patients and ethnic-matched healthy controls.

## 2. Materials and Methods

### 2.1. Study Population

AMI patients (*n* = 379, male 283, female 96, average age 60.43 years) were recruited from the Division of Cardiology, Affiliated Hospital of Jining Medical University (Jining, Shandong Province, China) during the period of April 2016 to April 2018. All AMI patients were diagnosed with clinical manifestations, electrocardiogram, and three-dimensional echocardiography. Ethnic-matched healthy controls (*n* = 386, male 198, female 188, median age 51.32 years) were from the Physical Examination Center in the same hospital. Controls with CAD family history were excluded from this study. This study was approved by the Human Ethic Committee, Affiliated Hospital of Jining Medical University, and conducted according to the principles of the Declaration of Helsinki. Informed consent was obtained from all participants.

### 2.2. Direct DNA Sequencing

Genomic DNAs were purified from peripheral blood leukocytes as previously described [22, 23]. The ATG5 gene promoter region (from −1069 bp to +103 bp to the transcription start site) was sequenced and analyzed. Two overlapped DNA fragments covering the ATG5 gene promoter, 649 bp (−1069 bp ∼ −421 bp) and 658 bp (−655 bp ∼ +103 bp), were generated by PCR. PCR primers were designed based on genomic sequence of the human ATG5 gene (NCBI, NC_000006.12) (Table 1). PCR products were bidirectionally sequenced with an Applied Biosystems 3500XL genetic analyzer. The sequences were aligned and compared with the wild-type ATG5 gene promoter, and genetic variants were identified.

### 2.3. Functional Analysis with Dual-Luciferase Reporter Assay

Report gene expression vectors with wild-type and variant ATG5 gene promoters were constructed with a firefly luciferase reporter vector (pGL3-basic). PCR primers to generate the ATG5 gene promoter are shown in Table 1. All expression vectors were further confirmed with sequencing. Designated expression vectors were then transfected into cultured cells (human embryonic kidney cells HEK-293 and rat cardiomyocyte cells H9c2), and dual-luciferase activities were examined. The experimental details were previously described [22, 23]. Transcriptional acitivity of the wild-type ATG5 gene promoter was designed as 100%. Relative activity of the variant ATG5 gene promoter was calculated. All the experiments were repeated three times independently, in triplicate.

### 2.4. Electrophoretic Mobility Shift Assay

Nuclear extract preparation and electrophoretic mobility shift assay (EMSA) was previously described [22, 23]. Nuclear extracts were prepared from HEK-293 and H9c2 cells. Double-stranded biotinylated oligonucleotides (30 bp) included wild-type (5′-TCCAACAAAGTAGAGAAGAAGATCAAATAA-3′) and genetic variant g.106326168_70delTCT (5′-TCCAACAAAGTAGAGAAGATCAAATAAGAA-3′).

### 2.5. Statistical Analysis

Quantitative data were expressed as mean ± SEM and analyzed by a standard Student's *t*-test. Frequencies of genetic variants in AMI patients and controls were analyzed and compared with SPSS v23.0. *P* < 0.05 was considered as statistically significant.

## 3. Results

### 3.1. Genetic Variants Identified in AMI Patients and Controls

**Figure 1:**
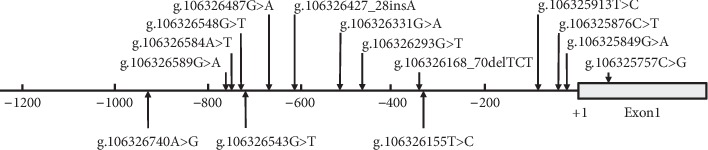
Locations and sequencing chromatograms of the genetic variants in the ATG5 gene promoter. The numbers represent the genomic DNA sequences of the human ATG5 gene (Genbank accession number NC_000006.12) upstream to the transcription start site (at the position of 106325820), which is set as +1.

**Figure 2:**
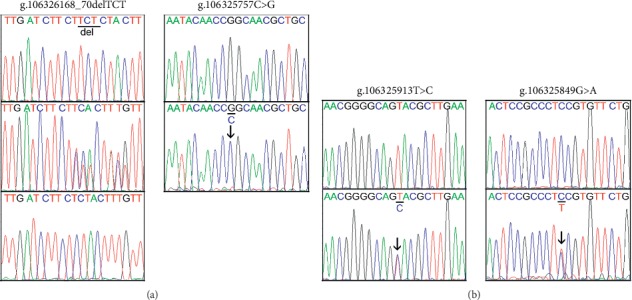
Sequencing chromatograms of the genetic variants in the ATG5 gene promoter. (a) Genetic variants in AMI patients. (b) Novel genetic variants in controls. For the variant (g.106326168_70delTCT), the top panel shows wild type, middle heterozygous, and bottom sequencing after cloning into a vector. For other genetic variants, top panels show wild-type DNA sequences and bottom panels show heterozygous variants, which are marked with arrows. For all variants, sequence orientation is forward.

A total of 15 genetic variants, including 6 single-nucleotide polymorphisms (SNPs), were identified in this study. Locations and frequencies of the genetic variants are depicted in Figure 1 and summarized in Table 2. A novel deletion variant (g.106326168_70delTCT) and an SNP [g.106325757C > G (rs190825454)] were only found in one 66-year-old male patient with non-ST-segment elevated AMI, and the sequencing chromatograms of which are shown in Figure 2(a). The SNP [g.106325757C > G (rs190825454)] was located in the untranslated exon 1 of the ATG5 gene. Two novel genetic variants (g.106325913T > C and g.106325849G > A) and three SNPs [g.106326548G > T (rs117781908), g.106326543G > T (rs182877945), and g.106326293G > T (rs560549742)] were only identified in controls. The sequencing chromatograms of the two novel variants are shown in Figure 2(b). Six novel variants (g.106326740A > G, g.106326584A > T, g.106326487G > A, g.106326427_28insA, g.106326331G > A, and g.106325876C > T) and two SNPs [g.106326589G > A (rs506027) and g.106326155T > C (rs510432)] were found in both AMI patients and controls with similar frequencies. Interestingly, two SNPs [g.106326589G > A (rs506027) and g.106326155T > C (rs510432)] were closely linked together with the same frequencies (*P* > 0.05).

#### 3.1.1. Genetic Variant-Related Putative Binding Sites for Transcription Factors

To determine whether genetic variant identified in AMI patients affected putative biding sites for transcription factors, the ATG5 gene promoter was analyzed with the JASPAR program (http://jaspar.genereg.net/). The variant g.106326168_70delTCT was predicted to abolish the binding sites for the Spi-B transcription factor (SPIB) and GATA binding protein 6 (GATA6), create the binding sites for the homeodomain factor RAX and transcription factor 7-like 2 (TCF7L2), and modify the binding sites for GATA2 and GATA3. As the SNP [g.106325757C > G (rs190825454)] was located in the untranslated exon 1 of the ATG5 gene, it was not analyzed.

### 3.2. Functional Analysis of the DSVs by Dual-Luciferase Reporter Assay

**Figure 3:**
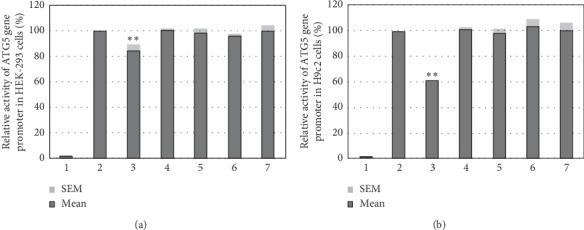
Relative transcriptional activity of wild-type and variant ATG5 gene promoters. Wild-type and variant ATG5 gene promoters were cloned into the reporter gene vector pGL3 and transfected into cultured cells. The transfected cells were collected, and dual-luciferase activities were assayed. Empty vector pGL3-basic was used as a negative control. Transcriptional acitivity of the wild-type ATG5 gene promoter was designed as 100%. Relative activities of ATG5 gene promoters were calculated. (a). Relative activities of wild-type and variant ATG5 gene promoters in HEK-293 cells. (b). Relative activities of wild-type and variant ATG5 gene promoters in H9c2 cells. Lanes 1, pGL3-basic; 2, pGL3-WT; 3, pGL3-106326168_70delTCT; 4, pGL3-g.106326293T; 5, pGL3-106325913C; 6, pGL3-106325876T, and 7, pGL3-106326155C. ^*∗∗*^, *P* < 0.01.

Activity of the wild-type and variant ATG5 gene promoter was examined in cultured cells by the dual-luciferase reporter assay (Figure 3). Expression vectors included empty pGL3-basic (negative control), pGL3-WT (the wild-type ATG5 gene promoter), pGL3-106326168_70delTCT, pGL3-g.106326293T, pGL3-106325913C, pGL3-106325876T, and pGL3-106326155C. In both HEK-293 and H9c2 cells, deletion variant (g.106326168_70delTCT) found in an AMI patient significantly decreased the activity of the ATG5 gene promoter (*P* < 0.01). In contrast, the SNP [g.106326293G > T (rs560549742)] and genetic variant (g.106325913T > C), which were identified in controls, did not significantly affect the activity of the ATG5 gene promoter in both HEK-293 and H9c2 cells (*P* > 0.05). As expected, the SNP [g.106326155T > C (rs510432)] and genetic variant (g.106325876C > T), which were found in both AMI patients and controls, did not significantly alter the activity of the ATG5 gene promoter (*P* > 0.05). Collectively, the deletion genetic variant (g.106326168_70delTCT) may change the transcriptional activity of the ATG5 gene promoter, which was not tissue-specific. In addition, as the SNP [g.106325757C > G (rs190825454)] was located in the untranslated exon 1 of the ATG5 gene, its effect on the ATG5 gene promoter was not examined.

#### 3.2.1. The Binding for Transcription Factors Affected by Genetic Variants

**Figure 4:**
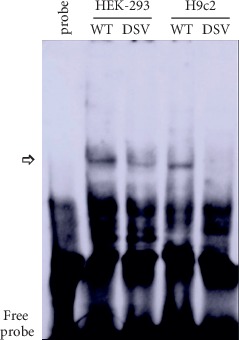
EMSA of biotin-labeled oligonucleotides containing the variant (g.106326168_70delTCT). Wild-type and variant oligonucleotides (30 bp) were designed and labeled with biotin. EMSA was conducted with biotinylated oligonucleotides and the nuclear extracts from HEK-293 and H9c2 cells. The free probe was marked with an arrow at the bottom. The affected binding for the transcription factor was marked with an open arrow.

To experimentally confirm whether the deletion genetic variant (g.106326168_70delTCT) affected the binding sites for transcription factors, EMSA was performed with nuclear extracts from HEK-293 cells and H9c2 cells. Double-stranded biotinylated oligonucleotides (30 bp) included wild-type (5′-TCCAACAAAGTAGAGAAGAAGATCAAATAA-3′) and genetic variant g.106326168_70delTCT (5′-TCCAACAAAGTAGAGAAGATCAAATAAGAA-3′). The results showed that the deletion genetic variant evidently weakened the binding of a transcription factor in both HEK-293 and H9c2 cells. Combined with the data predicted by the JASPAR program, further work will be needed to identify the transcription factor (Figure 4).

## 4. Discussion

Genetic variants in the ATG5 gene have been associated with several human diseases. An intron SNP in the ATG5 gene (rs9373839) has been associated with systemic sclerosis [24]. An intron SNP (rs12201458) and a promoter SNP (rs510432) are implicated in childhood asthma [25]. Intron SNPs in the ATG5 gene (rs573775 and rs665791) and the SNPs in the PRDM1-ATG5 intergenic region (rs548234 and rs6937876) confer to the susceptibility of developing systemic lupus erythematosus in different populations [26–29]. An intron SNP in the ATG5 gene (rs9372120) has been associated to multiple myeloma by a genome-wide association study [30]. In this study, we found a deletion genetic variant in the ATG5 gene promoter (g.106326168_70delTCT) and an SNP [g.106325757C > G (rs190825454)] in untranslated exon 1 of the ATG5 gene in one AMI patient. Furthermore, the deletion genetic variant significantly decreased the transcriptional activity of the ATG5 gene promoter by affecting the binding of a transcription factor. Therefore, the genetic variant in the ATG5 gene promoter may affect its transcription activity and change ATG5 levels, contributing to the AMI development as a rare risk factor.

The human ATG5 gene has been mapped to 6q21 [31]. It is transcribed into the mRNAs of 3.3 kb, 2.5 kb, and 1.8 kb at comparable levels in viable cells [32]. Though the ATG5 gene promoter has not been characterized, studies have demonstrated that the human ATG5 gene is regulated by transcription factors, microRNAs, and long noncoding RNAs. For example, P73 binds to and activates the human ATG5 gene promoter and regulates hepatocellular lipid metabolism [33]. NFE2L2 (nuclear factor, erythroid 2-like 2) activates ATG5 gene expression by regulating an enhancer antioxidant response element [34]. WNT5A overexpression leads to a significant increase in ATG5 mRNA levels in human melanoma cells [35]. miR-181A regulates the ATG5 gene through a functional mi-R181A responsive sequences in ATG5 3′ UTR in human cells [36]. In hepatocellular carcinoma, ATG5 is a direct target of miR-30b and an indirect target of long noncoding RNA HNF1A-AS1 [37]. Knockdown of lncRNA DICER1-AS1 inhibits ATG5 protein levels in human osteosarcoma cells [38]. Altered levels of ATG5 have been associated with human diseases, including multiple sclerosis, Alzheimer's disease, and cancers [39–41]. In this study, the deletion genetic variant (g.106326168_70delTCT) may change the activity of the ATG5 gene promoter by modifying the binding of a transcription factor.

ATG5 is a critical core protein for autophagosome formation and autophagic function [11]. In mice with ATG5 gene deletion, defective autophagy is observed in different tissues [15]. Moreover, tissue-specific ATG5 deficiency in cardiomyocytes or neural cells in mice causes tissue-specific autophagy loss and suppression [16, 17, 42]. Overexpression of the ATG5 gene in mice activates autophagy [18]. Accumulating evidence has demonstrated that ATG5-dependent autophagy regulates lipid metabolism and inflammation [4–6, 19, 43]. Recent studies have revealed many nonautophagic functions of ATG5, including autophagy-dependent cell death, cell proliferation, exosome secretion, granule exocytosis, immunological memory, LC3-associated phagocytosis, no-canonical protein secretion, pathogen control, and vision cycle [44, 45]. Particularly, LC3-associated phagocytosis as a novel function for autophagy proteins contributes to immune regulation and inflammatory responses [46, 47]. ATG5 regulates secretion of mature interleukin 1 beta and prevents its degradation [48, 49]. Collectively, the altered ATG5 level may influence lipid metabolism and inflammation through its autophagic and nonautophagic function, contributing to atherosclerosis and its complications.

## 5. Conclusions

In this study, we found a novel and functional deletion genetic variant in the ATG5 gene promoter in an AMI patient, but in none of controls. The genetic variant significantly altered the transcriptional activity of the ATG5 gene promoter likely by affecting the binding of a transcription factor. Therefore, this genetic variant may change the ATG5 level, contributing to AMI development as a rare risk factor.

## Figures and Tables

**Table 1:** PCR primers for the human ATG5 gene promoter.

PCR primers	DNA sequences	Location	Position	Products
Sequencing				
ATG5-F1	5′-GGCATGCTTCCCTAACTTGA-3′	106326889	−1069 bp	649 bp
ATG5-R1	5′-CCCACCCATCCAAGAGTACA-3′	106326241	−421 bp	
ATG5-F2	5′-TCTCGATCTCCTGACCTCGT-3′	106326375	−655 bp	658 bp
ATG5-R2	5′-CACTTCCGCCCTCTGGTAT-3′	106325718	+103 bp	
Functioning				
ATG5-F	5′-(KpnI)-GGCATGCTTCCCTAACTTGA-3′	106326889	−1069 bp	1172 bp
ATG5-R	5′-(HindIII)-CACTTCCGCCCTCTGGTAT-3′	106325718	+103 bp	

PCR primers are designed based on the genomic DNA sequence of the ATG5 gene (NC_000006.12). The transcription start site is at the position of 106325820 (+1).

**Table 2:** Genetic variants in ATG5 gene promoters in AMI patients and controls.

Genetic variants	Genotypes	Location^1^	Controls (*n* = 386)	AMI (*n* = 378)	*P* value
g.106326740A > G	TC	−920 bp	2	3	1.000
g.106326589G > A (rs506027)	GG	−769 bp	83	78	0.883
	GA		177	180	
	AA		126	120	
g.106326584A > T	AT	−764 bp	3	5	1.000
g.106326548G > T (rs117781908)	GT	−728 bp	1	0	—
g.106326543G > T (rs182877945)	GT	−723 bp	1	0	—
g.106326487G > A	GA	−667 bp	1	2	1.000
g.106326427_28insA	-/A	−607 bp	1	1	1.000
g.106326331G > A	GA	−511 bp	1	2	1.000
g.106326293G > T (rs560549742)	GT	−473 bp	1	0	—
g.106326168_70delTCT	TCT/-	−348 bp	0	1	—
g.106326155T > C (rs510432)	TT	−335 bp	83	78	0.883
	TC		177	180	
	CC		126	120	
g.106325913T > C	TC	−93 bp	1	0	—
g.106325876C > T	CT	−56 bp	1	1	1.000
g.106325849G > A	GA	−30 bp	1	0	—
g.106325757C > G (rs190825454)	GG	+69 bp	0	1	—

^1^Genetic variants are located upstream (-) to the transcription start site of the ATG5 gene at 106325820 of NC_000006.12.

## Data Availability

The data used to support the findings of this study are available from the corresponding author upon request.
